# A New Alternative Tool to Analyse Glycosylation in Monoclonal Antibodies Based on Drop-Coating Deposition Raman imaging: A Proof of Concept

**DOI:** 10.3390/molecules27144405

**Published:** 2022-07-09

**Authors:** Sabrina Hamla, Pierre-Yves Sacré, Allison Derenne, Ben Cowper, Erik Goormaghtigh, Philippe Hubert, Eric Ziemons

**Affiliations:** 1 Laboratory of Pharmaceutical Analytical Chemistry, Department of Pharmacy, University of Liege (ULiege), CIRM, Vibra-Sante Hub, 4000 Liege, Belgium; pysacre@uliege.be (P.-Y.S.); ph.hubert@uliege.be (P.H.); eziemons@uliege.be (E.Z.); 2 Center for Structural Biology and Bioinformatics, Laboratory for the Structure and Function of Biological Membranes, ULB, Campus Plaine CP206/02, 1050 Brussels, Belgium; allison.derenne@spectralysbiotech.com (A.D.); erik.goormaghtigh@ulb.be (E.G.); 3 National Institute for Biological Standards and Control, Blanche Lane, South Mimms, Potters Bar, Hertfordshire EN6 3QG, UK; ben.cowper@nibsc.org

**Keywords:** monoclonal antibodies (mAbs), drop-coating deposition Raman imaging (DCDR), multivariate curve resolution-alternating least square (MCR-ALS), singular value decomposition (SVD), nonlinear support vector regression (SVR), P-vector

## Abstract

Glycosylation is considered a critical quality attribute of therapeutic proteins as it affects their stability, bioactivity, and safety. Hence, the development of analytical methods able to characterize the composition and structure of glycoproteins is crucial. Existing methods are time consuming, expensive, and require significant sample preparation, which can alter the robustness of the analyses. In this context, we developed a fast, direct, and simple drop-coating deposition Raman imaging (DCDR) method combined with multivariate curve resolution alternating least square (MCR-ALS) to analyze glycosylation in monoclonal antibodies (mAbs). A database of hyperspectral Raman imaging data of glycoproteins was built, and the glycoproteins were characterized by LC-FLR-MS as a reference method to determine the composition in glycans and monosaccharides. The DCDR method was used and allowed the separation of excipient and protein by forming a “coffee ring”. MCR-ALS analysis was performed to visualize the distribution of the compounds in the drop and to extract the pure spectral components. Further, the strategy of SVD-truncation was used to select the number of components to resolve by MCR-ALS. Raman spectra were processed by support vector regression (SVR). SVR models showed good predictive performance in terms of RMSECV, R^2^_CV_.

## 1. Introduction

The development of protein-based drugs has accelerated in recent years as proteins exhibit a variety of therapeutically beneficial properties, such as: higher target specificity and generally fewer side effects. Most drugs derived from biotechnology marketed to date are derived from recombinant proteins. Recombinant proteins represent the majority of biopharmaceuticals on the market. These include hormones, cytokines, enzymes, and monoclonal antibodies, which have rapidly grown thanks to their effectiveness in the treatment of severe diseases.

Glycosylation is present in almost 60% of commercial pharmaceutical proteins and is considered as the most common post-translational modification (PTM) of extracellular proteins and some intracellular proteins [1]. The characterization and quantification of glycosylation have always been considered as a challenging task in the analysis of biopharmaceuticals, due to the structural complexity of glycans. This complexity is mainly due to the variable composition, linkage, and branching of the monosaccharides in glycans [2,3]. 

Moreover, throughout the manufacturing process, glycosylation can be influenced by many parameters, such as the host system type (mammalian cells, yeast strains, plant cells, insect cells, or genetically modified animals) and environmental culture conditions (bioreactor type, culture media, and process parameters). Although these parameters are monitored and controlled throughout the development and manufacturing process, this does not prevent the occurrence of micro- and macro-heterogeneity in the glycosylation of proteins. Heterogeneity of protein glycosylation profile may significantly affect the quality and the safety of the final therapeutic product [4].

Consequently, the regulatory authorities require systematic characterization of the composition and structure of glycoproteins throughout the drug development and manufacturing processes. Existing glycosylation analysis methods can be time consuming, expensive, and involve significant sample preparation steps, which can lead to several sources of error and, therefore, alter the robustness of the analyses. The sample preparation protocols generally require a minimum of three steps: glycan release, labelling, and purification [5]. The development of direct and simple methods may, therefore, improve the monitoring or characterization of glycans [1,6].

In pharmaceutical and biological applications, confocal Raman microscopy has the advantage of high spatial resolution. It is an important tool with a high content of chemical information [7]. However, in an aqueous solvent containing buffering agents, the Raman scattering of these buffering agents can overwhelm the Raman scattering of compounds, which are present at lower concentrations [8]. In this study, a fast, direct method based on drop-coating deposition Raman imaging (DCDR) combined with a chemometrics approach is proposed to analyze glycosylation in monoclonal antibodies (mAbs). The DCDR method simply consists of depositing a small volume of solution on a hydrophobic substrate and allowing it to dry for a few minutes. Indeed, as the solvent evaporates, a “coffee ring” is formed, delimiting the central zone, which retains the buffering agents from the edge, which is concentrated in protein. This method has shown good efficiency in the separation of biopolymers, such as proteins, as well as highly water-soluble compounds, such as buffers. Further, this method overcomes both fluorescence issues if present and spectral interferences from buffer and other compounds present in the solution [7]. In the literature, the study of Barman et al. demonstrated the potential of the DCDR technique, as a new analytical method for selective detection and quantification of glycated hemoglobin HbA1c in the context of clinical chemistry (treatment of diabetic patients) [9].

The objective of this work is twofold. First, the study aims at demonstrating the efficiency of DCDR imaging to analyze pharmaceutical products of monoclonal antibodies (mAbs). Therefore, in order to obtain the spatially resolved chemical images (scores) and the corresponding resolved Raman spectra of individual chemical species (pure components), MCR-ALS was used to unmix the original series of spectra present in the hyperspectral images. In this context, the determination of the number of components involved in the studied process is an important step to perform in the MCR-ALS analysis [10]. Indeed, the wrong choice of the number of components, can generate an overestimation of the rank in the matrix; thus, MCR-ALS leads to the extraction of components not representative of the chemical reality. Further, an underestimation of the rank can occur and, thus, make a complete characterization of the sample impossible and, thus, result in a loss of information. This is the reason why, in this work, the singular value decomposition (SVD truncation strategy) was used to allow the determination of the number of components to be selected in the MCR-ALS and also to noise the Raman maps. The application of the SVD does not require any prior knowledge of the raw data. The goal is to find the most relevant chemical information to avoid rank deficiencies in MCR-ALS. Indeed, this algorithm allows one to have an efficient truncation of the information in the data. Therefore, only the singular values and singular vectors relevant from a chemical point of view are selected [11]. Second, the study aims at quantifying the relative amount of each major monosaccharide (mannose, N-acetylglucosamine, galactose, fucose, and sialic) and glycans (M5, FA2G2, sialylated glycan, high mannose). The pure spectral component of protein resolved by MCR-ALS is used in the regression model as input spectral data. Several regression models were calibrated to determine the relative amount of each monosaccharide and glycan. A hyperspectral Raman dataset of 16 reference mAbs samples was collected. The composition in glycans and monosaccharides was determined using reference LC-FLR-MS methods.

Support vector regression (SVR) was established to correlate the spectral information (pure component) and the quantitative reference values. This regression was chosen as it can cope with high degrees of nonlinearity. Moreover, it is a robust method, which is less sensitive to spectral noise and provides accurate predictions [1]. SVR models provide less diagnostics in terms of specificity, because the information about the original input variables is lost [12]. This is why the study on the specificity of SVR models was also explored.

## 2. Materials and Methods

### 2.1. Chemicals, Reagents, and Proteins for the Spectral Characterization of Commercial mAbs Solutions

16 monoclonal antibodies (mAbs) were analyzed (one batch per product). These glycoproteins were provided by the Saint-Pierre hospital (Brussels, Belgium) and the pharmacy of the University Hospital Center of Liège (CHU Liège, Belgium).

The 16 mAbs and their producers are as follows:

Avelumab (Bavencio^®^ 20 mg mL^−1^, Merck, Lyon, France)

Bevacizumab (Avastin^®^ 25 mg mL^−1^, Roche Pharma, Basel, Swiss)

Cetuximab (Erbitux^®^ 5 mg mL^−1^, Merck KGaA, Darmstadt, Germany,)

Daratumumab (Darzalex^®^ 20 mg mL^−1^, Janssen Biologics, Leiden, Netherlands)

Durvalumab (Imfinzi^®^ 50 mg mL^−1^, AstraZeneca, Reims, France)

Infliximab (Remicade^®^ 10 mg mL^−1^, Janssen Biologics)

Ipilimumab (Yervoy^®^ 5 mg mL^−1^, Bristol Myer Squibb, New York, NY United States)

Nivolumab (Opdivo^®^ 10 mg mL^−1^, Bristol Myer Squibb)

Ocrelizumab (Ocrevus^®^ 1.2 mg mL^−1^, Roche, Grenzach-Wyhlen, Germany)

Panitumumab (Vectibix^®^ 25 mg mL^−1^, Amgen, Boulogne Billancourt, France)

Pembrolizumab (Keytruda^®^ 25 mg mL^−1^, Merck)

Pertuzumab (Perjeta^®^ 30mg mL^−1^, Roche Pharma)

Ramucirumab (Cyramza^®^ 10mg mL^−1^, Eli-Lilly, Brussels, Belgium)

Rituximab (Mabthera^®^ 10 mg mL^−1^, Roche Pharma)

Trastuzumab (Herceptin^®^ 21 mg mL^−1^, Roche Pharma)

Ustekinumab (Stelara^®^ 5 mg mL^−1^, Vidal, Issy-les-Moulineaux, France)

Excipients were purchased from Sigma-Aldrich (Merck, Bornem, Belgium) and were prepared at 10 mg mL^−1^ in a NaCl (0.9%) solution.

Sucrose

Polysorbate 20 and 80 

Sodium acetate

L-Histidine

Glycine

Mannitol

Monosodium phosphate

Disodium phosphate

Histidine hydrochloride

Trehalose

TRIS hydrochloride

Sodium citrate

Citric acid

### 2.2. Chemicals, Reagents, and Proteins Used to Study the Specificity of SVR Models

Monosaccharides were purchased from Sigma-Aldrich (Merck, Lyon, France) and Dextra* and were prepared at 10 mg mL^−1^ in NaCl (0.9%) solution:

Sialic acid

Galactose

Fucose

N-Acetylglucosamine

D-Mannose*

M5 Glycans were purchased from Dextra and were prepared at 1 mg mL^−1^ in NaCl (0.9%) solution. 

### 2.3. Solution Preparation

The stock solutions of the mAbs were analyzed at their initial concentration. Additionally, two aliquots of the stock solutions were prepared. A first aliquot was prepared in NaCl (0.9%) at 3 mg mL^−1^. A second aliquot was prepared by taking 50 µL of the stock solution. This second aliquot was eventually purified by column filtration.

#### 2.3.1. Detection Limits

To study the quantitative limits of detection (LoD) in DCDR analysis with the µ-RIM slide, 7 dilutions of each antibody were prepared and analyzed. 

The therapeutic concentration ranges were chosen based on the pharmaceutical preparations (CHU Liege). The different concentrations were prepared from the stock formulations using serial dilutions in NaCl (0.9%). Table 1 illustrates the different dilutions prepared for each commercial mAb solution. All samples were prepared freshly on the day of the analysis.

#### 2.3.2. Removal of Excipients in mAbs Formulations

**Figure 1:**
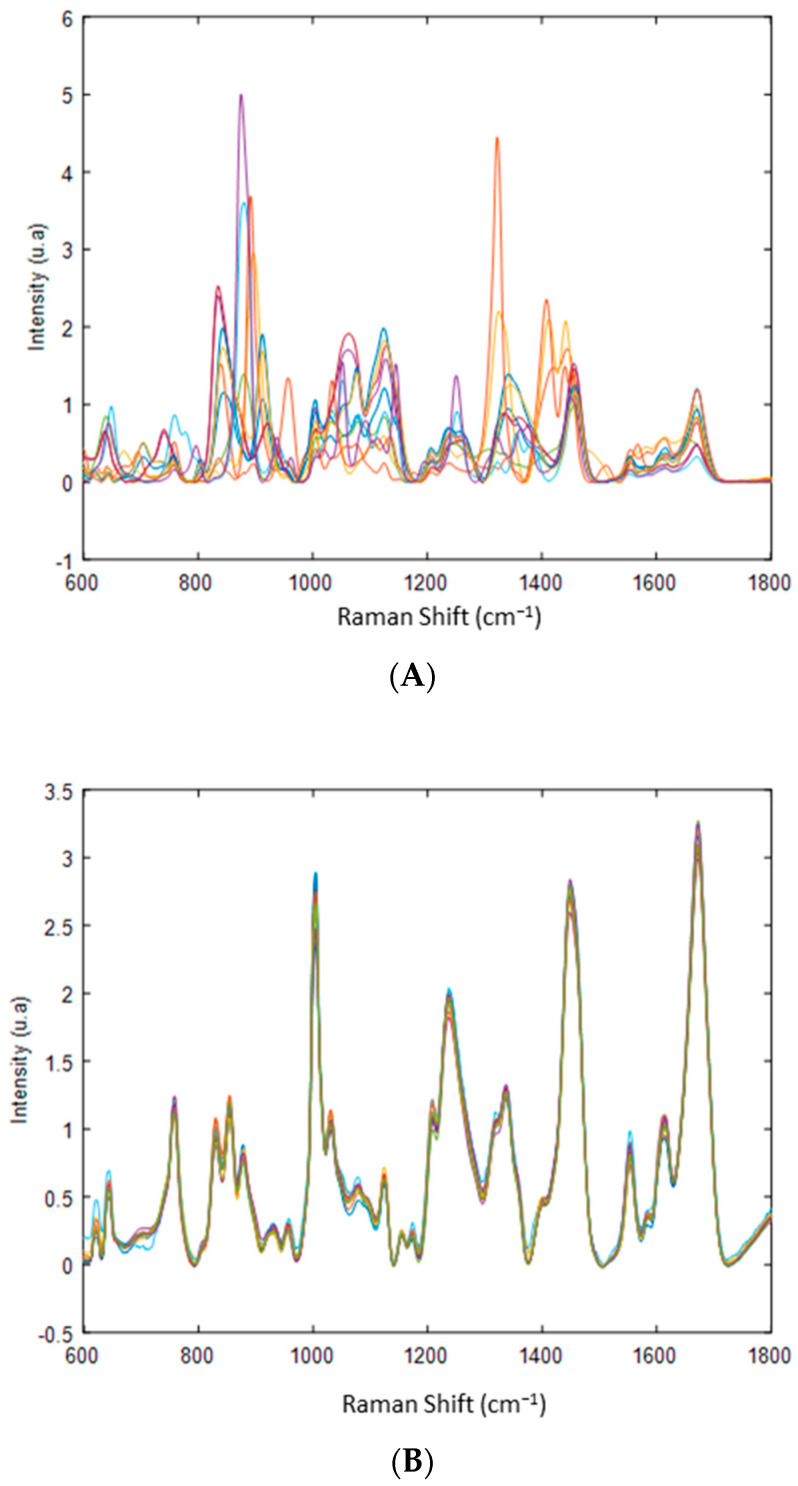
(**A**) Preprocessed Raman spectra of therapeutic proteins in their pharmaceutical formulation. (**B**) Preprocessed Raman spectra recorded after removal of residual salts and excipients present in the formulations of therapeutic proteins using size exclusion spin columns. Baseline correction and standard normal variate (SNV) were applied as preprocessing.

The study first aimed to demonstrate that the DCDR analysis allows the separation of the buffer salts, excipients, and glycoprotein. To check the efficiency of this separation, the spectral profile of the glycoprotein after purification via filtration column and via the DCDR method were compared. Size-exclusion spin columns were used to remove residual salts and excipients present in the formulations. These excipients (mannitol, Tween 80, Tween 20, trehalose, etc.…) interfere with Raman measurements in the spectral region between 600 and 1800 cm^−1^ as shown in Figure 1A,B. Furthermore, due to their polyol nature, they prevent the analysis of the glycan part of the mAbs.

As explained in [1], a buffer exchange step with NaCl (0.9%) was performed using Micro Bio-Spin^®^ P-6 Gel columns (TRIS buffer, sample volume 10–75 µL, 6000 Da MW limit). The size-exclusion spin columns are based on the principle of gel filtration (Polyacrylamide). Therefore, molecules larger than the pores of the stationary phase matrix will be first excluded and migrate rapidly through the column, while molecules smaller than the matrix pores migrate more slowly [13].

Thus, at the end of the filtration cycle, the solutions were recovered in 2 fractions: the concentrated proteins were first eluted and recovered in the filtrate, while the excipients were recovered from the filter after washing with the NaCl solution. To ensure efficient separation, three cycles were applied to each mAb solution [6].

### 2.4. Raman Analysis

**Figure 2:**
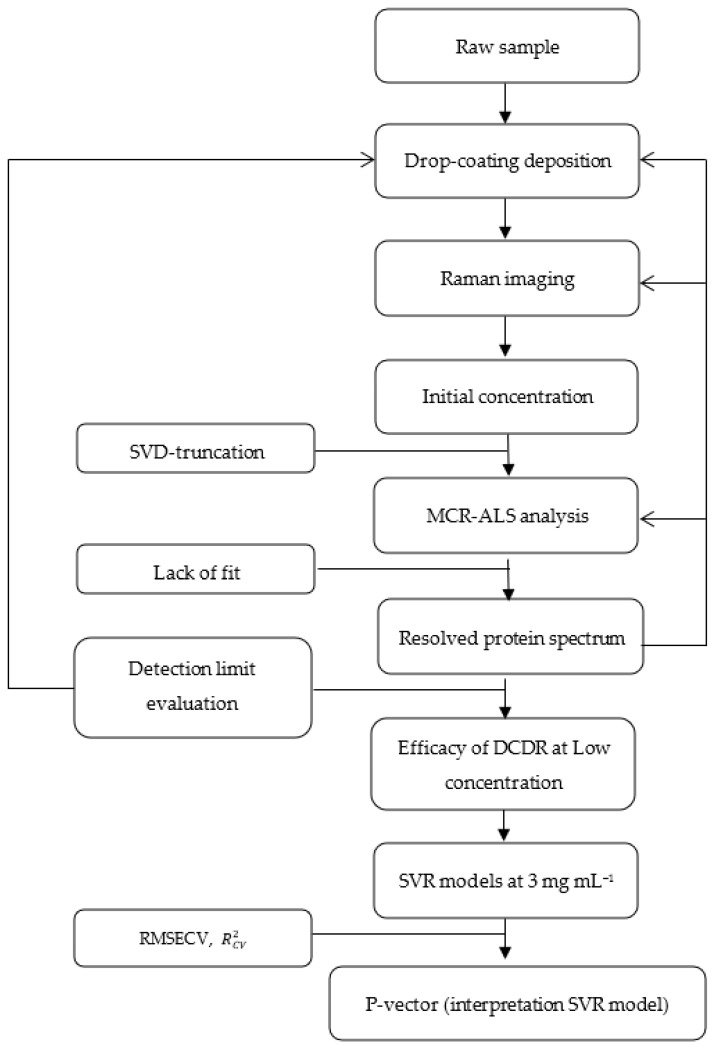
Schematic description of the general methodology followed in the study for the analysis of mAbs by drop-coating deposition Raman combined with MCR-ALS analysis and SVR.

Next, 2 μL of each sample was deposited on a µ-RIM (BioTools, Jupiter, JUP, United States) slide covered with a thin layer of polytetrafluoroethylene (PTFE) and dried at ambient temperature for 45 min. All Raman spectra and hyperspectral Raman imaging data were acquired with a Labram HR Evolution (Horiba scientific, Lyon, France) equipped with an EMCCD detector (1600 × 200 pixels, Andor Technology Ltd., Abingdon-on-Thames, UK.). A 600 gr/mm grating, a Leica 50× Fluotar LWD objective and a 532 nm laser with a power of 25 mW at the sample (Horiba Scientific) were used. Figure 2 illustrates the methodology followed throughout this study.

#### 2.4.1. Raman Spectral Measurements

All spectra were collected with the LabSpec 6 software (Horiba Scientific, Jobin Yvon, Palaiseau, France) over a spectral range 600–1800 cm^−1^ with 10 s acquisition time and two accumulations. Three spectra were acquired at four distinct positions of the dried drop for each sample: concentrated protein, filter of Micro Bio-Spin^®^ (excipients), and excipients. 

**Figure 3:**
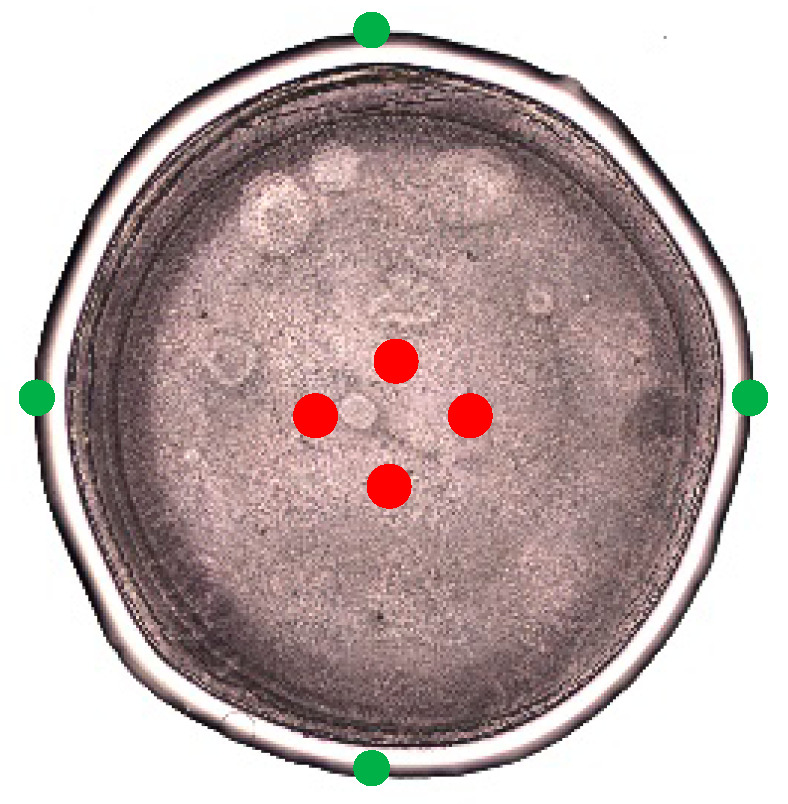
Illustration of the different positions of the Raman acquisition for the limit-of-detection (LOD) evaluation. The green spot corresponds to the measure of concentrated proteins and the red spot corresponds to the measure of excipients.

Regarding the limit of detection (LOD), a spectral range between 600 and 1800 cm^−1^ was used. For each concentration, three spectra were acquired at four distinct positions of the dried drop, on the edge and the center of the drop (Figure 3). 

Finally, for the interpretation of SVR models, the spectra of each of the five pure monosaccharides and the Man-5 glycan were measured. In this context, six spectra (six distinct deposits) were recorded for each sample.

#### 2.4.2. Raman Hyperspectral Imaging

For the composition analysis of the mAbs, 16 hyperspectral images were acquired. For the quantitative analysis of the composition in monosaccharides and glycans, a total of 96 hyperspectral images at a low concentration (3 mg mL^−1^) of mAbs were acquired. Two independent samples with 3 distinct deposits for each sample were acquired. 

A Leica 10× Fluotar LWD objective was used to map the sample’s surface. The spectra were acquired from 600 to 1800 cm^−1^. For the initial product concentrations of mAbs, a 10 s acquisition time and two accumulations were used, giving a total data acquisition time of 2 h 55 min. In order to reduce the analysis time, these parameters were reduced, without losing quality, because at low concentration, the mAbs are more concentrated at the edge of the drop. Therefore, low-concentration maps were acquired with 2 s acquisition time and two accumulations leading to 38 min.

Raman spectra were collected on the whole drop surface with 25 × 25 pixels mapping and a step size of 134 μm on the X and Y dimensions. The spectral grids were spherical in shape based on the dimensionality of the drop. 

### 2.5. Reference Analysis: LC-FLR-MS N-Glycans Characterization

#### 2.5.1. Glycoworks RapiFluor-MS N-Glycan

The Glycoworks RapiFluor-MS N-glycan 24 samples kit (#176003713) was purchased from Waters Corporation (Milford, MA, USA). This analytical method allows rapid deglycosylation followed by fluorescent labelling and purification of the labelled glycans [6]. 

#### 2.5.2. UPLC-FLR-MS Analysis

Labelled N-glycans were analyzed via HILIC separation combined with fluorescence (FLR) and mass spectrometry (MS) detection using a UPLC-MS system equipped with an ACQUITY UPLC BEH Amide (2.1 mm × 150 mm, 1.7 µm particle size, and 130 Å pore size) column (Waters Milford, MA, USA). The details of this method are described in Derenne et al. [6]. MS data were obtained using a Single Quadrupole Detector 2, SQD2 (Waters, Milford, MA, USA) in ESI-positive mode and the data were acquired using Empower 3.1 software. 

### 2.6. Multivariate Data Analysis

MCR-ALS analysis, PLSR, and SVR method were performed in MATLAB^®^ R2017b (The MathWorks, Inc., Natick, MA, USA) using PLS_Toolbox^®^ 8.2.1 (Eigenvector Research, Inc., Manson, WA, USA). Furthermore, SVD truncation strategy and P-vector were programmed under the MATLAB^®^ environment [12].

#### 2.6.1. Data Preprocessing

All Raman spectra were preprocessed by Savitzky and Golay smoothing (window size: 7) for noise reduction and followed by a baseline correction by the Automatic Whittaker filter (λ = 100,000, *p* = 0.001).

The resolved Raman spectra used to build the monosaccharide and glycan prediction models were preprocessed first, by Savitzky and Golay 1st derivative (polynomial order: 3, window size: 15) and followed by standard normal variate (SNV).

#### 2.6.2. Multivariate Curve Resolution Alternating Least Squares (MCR-ALS)

The hyperspectral Raman imaging data were analyzed using MCR-ALS to generate spatially resolved chemical images (scores) and corresponding resolved Raman spectra of the individual chemical species (pure components). This method is often used in the case of complex samples of unknown composition [14,15].

Each hyperspectral Raman imaging datum is a three-dimensional data cube $\underline{D}\;\left(x\times y\times \lambda \right)$, where x, y are the spatial information and λ is the spectral information (cm^−1^). In order to be able to carry out the MCR-ALS, $\underline{D}$ is unfolded as a 2D data matrix D (n×m), where n=x×y and m=λ. 

Mathematically, MCR-ALS decomposes the data matrix, D, as follows:

$$D=C.S^{T}+E$$

where C is the matrix of the relative concentration profiles, S the matrix of pure spectra, and E the matrix of the residuals. 

In this work, MCR-ALS was initialized via exteriorpts algorithm and non-negativity constraints were applied on the concentration and on the spectra [9]. The full spectral range (600–1800 cm^−1^) of the given hyperspectral Raman imaging dataset was used in MCR-ALS. 

In this study, the pure chemical components resolved for each mAb sample were used as input data for the regression models.

The lack of fit (LOF) was calculated to evaluate the quality of the fit of the MCR-ALS model.

$$LOF=\sqrt{\frac{\sum_{i=1}^{n}\sum_{j-1}^{m}(d_{i,j}-{\hat{d_{i,j})}}^{2}}{\sum_{i=1}^{n}\sum_{j-1}^{m}{\left(d_{i,j}\right)}^{2}}}\times 100$$

where:

- $d_{i,j}$ corresponds to the input original data.
- $d_{i,j}-\hat{d_{i,j}}$ corresponds to the residual calculated from the difference between the input original data and the MCR-ALS reproduction.

#### 2.6.3. Singular Value Decomposition (SVD Truncation Strategy)

The estimate for the number of chemical species (rank) is necessary in order to carry out the MCR-ALS analysis. Different analysis methods exist to estimate this rank, such as principal component analysis (PCA), Durbin–Watson (DW) criterion, and the singular value decomposition (SVD) method. In linear algebra, the rank corresponds to the number of pure spectra (eigenvectors) or concentration profiles necessary to explain the set of recorded data, the latter being only a linear combination of these eigenvectors. In the ideal case, in the absence of measurement noise, the chemical rank corresponds to the definition of the mathematical rank [16].

In this study, SVD (truncation strategy) was applied first to denoise the hyperspectral Raman imaging data and secondly to estimate the number of chemical species in the MCR-analysis.

- The first step of the SVD is a factorization of the data matrix D (n×m) as
  $$D=U\Sigma V^{T}$$
  where:
  U (n×n) and V (m×m) are left and right singular vectors matrices, respectively.
  Σ(n×m) is the diagonal matrix of the singular values $\sigma_{i}$ for i=1,…,r with r being the rank of the matrix D.
- The second step consists of plotting the singular values according to their value. Thus, the first, second, and third segment are identified. Only the second (between two breaks) is considered since it represents a good compromise between the relevant information carried by the segment on the left and the noise carried by the segment on the right. Each $\sigma_{i}$ of this segment is considered as a rank and then as a threshold.
- The last step consists of a reconstruction of the different truncated matrices (different ranks r) $\hat{D}\;\left(n\times m\right)$ like $\hat{D}=\hat{U}\hat{\Sigma }{\hat{V}}^{T}$. In this study, 3 truncated matrices were considered.

For each of these matrices, an MCR model is built and analyzed. Only the interpretable model, related to the best estimate of the rank, was retained. 

#### 2.6.4. Performance Evaluation of the SVR Models

Different spectral ranges were tested to build the models. The best spectral range was selected based on its cross-validation performance. As a result, the spectral range between 800 and 1115 cm^−1^ was retained. It is also in this spectral range that the characteristic bands of sugars are present [17].

To calibrate and optimize SVR models, Venetian blinds was used as a cross-validation strategy with a data split of 10 and one sample per blind (thickness). In this context, the calibration models were evaluated using the Root Mean Square Error of cross-validation (RMSECV).

$$RMSECV=\sqrt{\frac{\sum_{i=1}^{n}{\left({\hat{y}}_{CV,i}-y_{i}\right)}^{2}}{n}}$$

$$R_{CV}^{2}=\sqrt{1-\frac{\sum_{i-1}^{n}{\left({\hat{y}}_{CV,i}-y_{i}\right)}^{2}}{\sum_{i=1}^{n}{\left({\hat{y}}_{CV,i}-\bar{y}\right)}^{2}}}$$

where ${\hat{y}}_{CV,i}$ is the value predicted by the cross-validated model for sample *i*. $y_{i}$ is the measured value obtained for sample *i* and *n* is the number of samples. $\bar{y}$ corresponds to the average of all reference measurement values in the calibration set [18].

Thereby, an evaluation of performances for the SVR models was performed, by comparing the results obtained by the calibration performances: RMSECV and $R_{CV}^{2}$. Low value of RMSECV and a high value of $R_{CV}^{2}$ are expected indicating that the model is able to accurately estimate the concentration.

#### 2.6.5. Interpretation of SVR Models

Gaussian RBF kernel (Radial Basis Function) was chosen to perform the SVR models. This kernel expresses sample-to-sample similarities using the following equation:

$$K\left(x_{i},x_{j}\right)=\exp(-\gamma \|x_{i}-x_{j}\|{}^{2}),$$

where γ>0 and $x_{i\;},x_{j}$ are input feature values for *i* and *j* samples and γ is the kernel parameter. The nonlinear SVR model requires the determination of three meta-parameters: the cost C, and the variables ε and γ. To have a good generalization performance of SVR models, the simultaneous optimization of its three meta-parameters (C, ε and γ) was performed. This optimization is conducted through a grid search using 2-step cross-validation. The first step was a coarse grid search in a goal to select approximately the best region. The second step was a finer grid search in order to obtain optimal values. 

Consider an input dataset X(N×M) with an output vector $y_{i}\in R$. The objective of SVR is to find a multivariate regression function f(x) to predict the desired output property (amount of monosaccharides) of an unknown object (new spectrum).

(1) $$f\left(x\right)=\sum_{i,j=1}^{N}\left(\alpha_{i}-\alpha_{i}^{*}\right)K(x_{i},x_{j})+b$$

where $\alpha_{i}\;\mathrm{and}\;\alpha_{i}^{*}$ correspond to the support vectors, $\alpha_{i},\alpha_{i}^{*}\neq \;0$.

**Figure 4:**
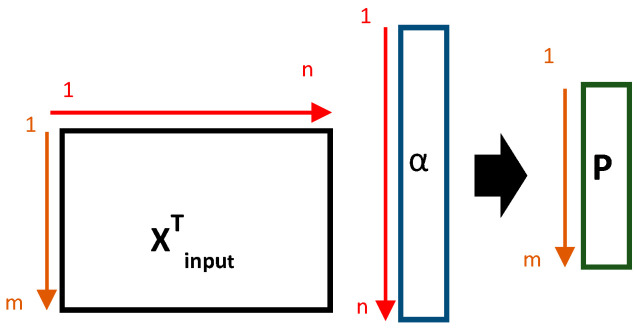
The P-vector is obtained by the inner product of **X**^T^ and the α vector of the SVR model.

$K\left(x_{i},x_{j}\right)$ is an RBF kernel that transforms the nonlinear input space into a high-dimensional feature space, where the problem can be modeled in a linear way. However, the information related to the original input variables is lost. Therefore, it is important to determine the contribution of each input variable to the final regression model to ensure the specificity of the model. Thus, being able to clearly determine which input variables in the original input data is explanatory for the modeled output property (amount of monosaccharides). Therefore, a P-vector is obtained by calculating the inner-product between the original input space $X^{T}$ and the α-vector of the SVR models (Figure 4) [2].

## 3. Results and Discussion

### 3.1. Spectral Characterization of Commercial mAbs Solutions after Excipients Removal

Sixteen commercial mAbs listed in Table 2 were filtered by Microbiospin and the filtrate and filter were recovered and analyzed by Raman.

- Analysis Filtrate: concentrated mAbs

**Figure 5:**
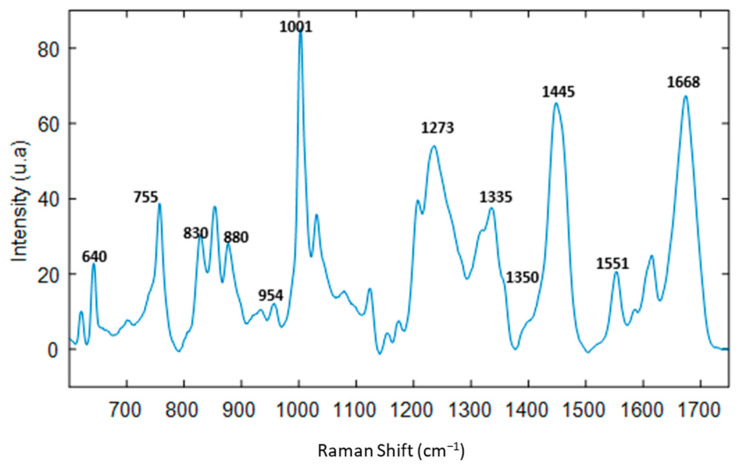
Mean Raman spectrum of 16 mAbs after filtration of the excipients, acquired by depositing drops after 45 min of drying.

Figure 5 shows the average Raman spectrum of 16 mAbs after filtration of the excipients, acquired by depositing drops after 45 min of drying. Therefore, the main feature bands of proteins are observed at ~640 cm^−1^ (tyrosine), 755 cm^−1^ (tryptophan), 880 cm^−1^ (C-C backbone), 954 cm^−1^ (C-H stretching of α-helix structure), 1001 cm^−1^ (phenylalanine), 1237 cm^−1^ (amide III), 1335 cm^−1^ and 1445 cm^−1^ (CH2 deformation), 1551 cm^−1^ (amide II), and 1668 cm^−1^ (amide I). Notably, the main feature bands of sugar are observed at 880 cm^−1^ (C-O-C stretch) and 1350 cm^−1^ (NH2 twist), and the bands of glycosidic ring are observed at (830, 1000, 1450 cm^−1^) [17,19].

- Analysis filter: excipients

Figure S1 reports the analysis on the filter retained during the filtration of trastuzumab. Table 2 shows the detected spectrum of each commercial mAbs solution, after superposition of the spectrum retained in the filter with each of the excipients present in the formulation of mAbs. It is interesting to note that the dominant spectrum corresponds to the excipient at the highest concentration in the sample (Table S1), whilst the other excipients present at very low concentration are not detected [20]. 

### 3.2. Spectral Characterization of Commercial mAbs Solutions by DCDR Imaging and MCR-ALS Analysis

In previous work, we proposed to use ATR FT-IR spectroscopy to qualitatively and quantitatively analyze the glycosylation of monoclonal antibodies [1]. However, this method has some limitations; for example, it requires protein deposits with uniform chemical composition, and to obtain a high-quality ATR spectrum, approximately 300 μg mL^−1^ of protein is required [21]. In addition, a preliminary step of elimination of excipients by specific filtration columns is necessary. Therefore, this study, using the DCDR method, presents some advantages, which overcome the limitations previously encountered with the FTIR-ATR method. Indeed, it requires a microdeposition of only 10 ng of protein to obtain high-quality spectra. Further, the excipient removal step, which was previously mandatory, is no longer necessary.

In this context, the first aim of this study was the identification of composition of mAbs in their pharmaceutical formulation. For this, the use of a hydrophobic substrate is beneficial for the formation of the coffee ring. Several hydrophobic substrates have already been used since the development of the DCDR method, including quartz, CaF_2_, polished steel with a thin coating of polytetrafluoroethylene (PTFE), etc. In this study, slides of PTFE-coated steel surfaces were used. PTFE-coated slides are more advantageous than slides coated with CaF_2_, which lose their hydrophobicity over time [22].

This method is based on physical segregation and requires only the deposition of a drop of solution on this specific substrate and, after a few moments, the solvent is evaporated. Thus, the separation of excipients and protein was achieved by forming the “coffee ring” [8]. Further, in this study, the use of hyperspectral imaging enabled the visualization of the distribution of the compounds in the drop.

**Figure 6:**
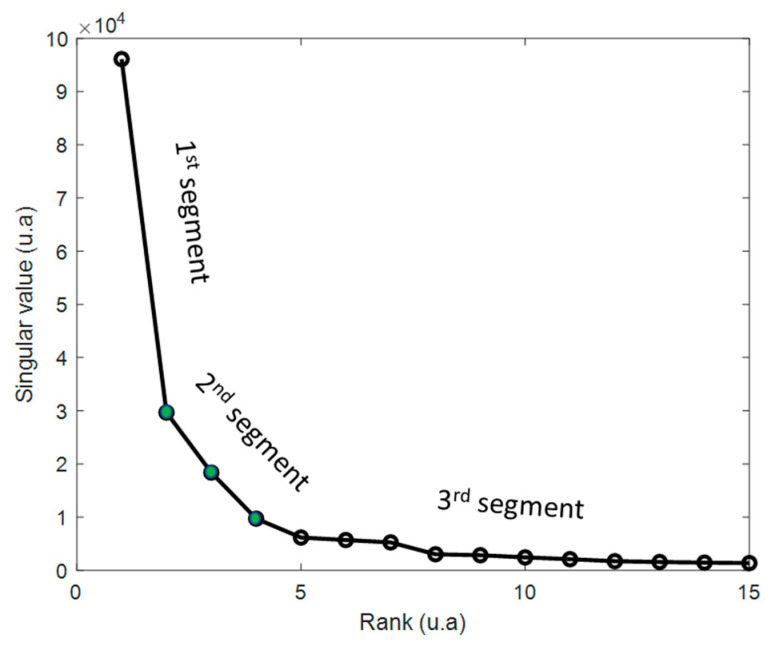
Singular values reported according to their rank, which shows three segments. Three thresholds (green dots) were considered in the second segment and, thus, three reconstructions of the different truncated matrices (different ranks r) were performed.

Rank estimation (number of pure spectral signatures) for the MCR-ALS analysis was obtained based on the SVD truncation strategy. The rank is estimated when the singular values are zero. In our case (Figure 6), the singular values decrease but do not reach zero. It is, therefore, not possible to give an exact value of the rank. This is why the notion of threshold is used to determine the significant singular values with respect to the variance in the noise [23]. As a result, three segments are identified; the first segment contains only information, the third segment contains only noise, and the second segment contains a mixture of information and noise. Thus, the last step consists of the selection of three ranks of this second segment. Finally, the reconstruction of three truncated matrices is performed, then they are introduced into the MCR-ALS in order to choose the correct reconstruction. 

**Figure 7:**
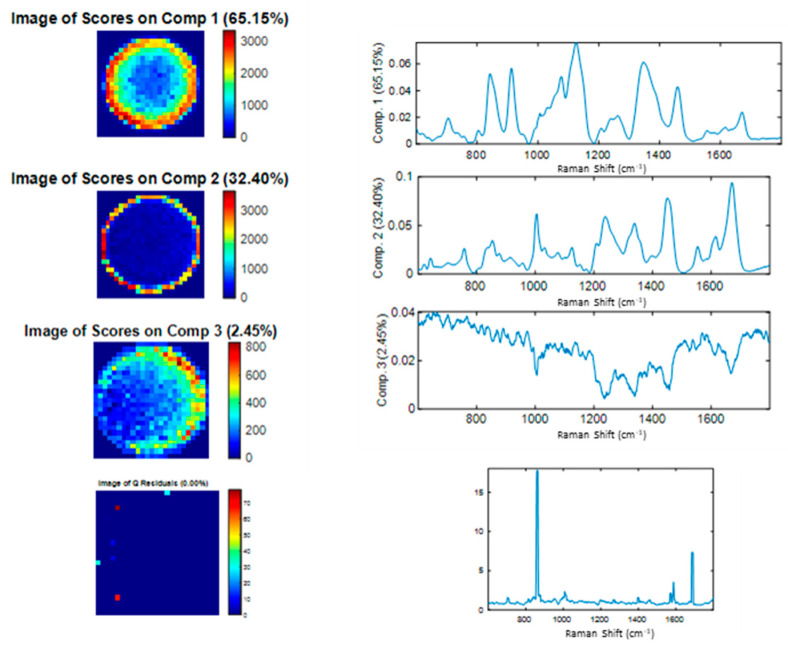
MCR-ALS analysis of trastuzumab at initial product concentration, illustrating the three pure components extracted from the MCR-ALS, after application of SVD, as well as the image of the scores, which are displayed in color code, according to the intensity of the information, from the less intense (blue color) to the more intense (red color).

**Figure 8:**
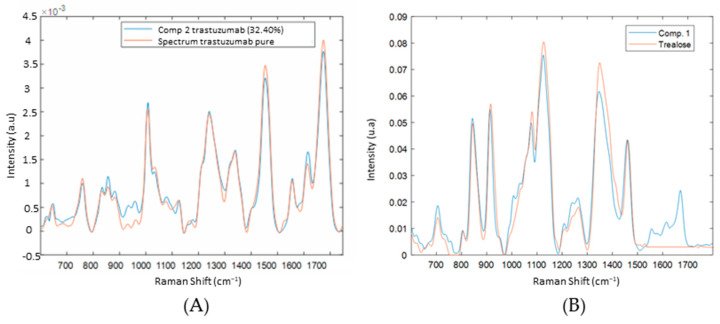
(**A**) Superimposition of the Raman spectrum, acquired at the outer edge of the drop with the spectrum of the protein purified with the Microbiospin^®^. (**B**) Superimposition of the Raman spectrum, acquired at the center edge of the drop with the spectrum of the excipient (trehalose).

Figure 7 shows the application of the DCDR method combined with MCR-ALS analysis on the trastuzumab analysis. In this example, the selected number of components determined by SVD-truncation is three. The drying process of the drop allowed the separation of solutes in the solution due to the formation of a “coffee ring” [2]. Therefore, in Figure 7, component 1 represents the spectrum of the excipient with weak protein bands (amide I, II, III), located in the inside edge of the drop. Component 2 represents the spectrum of the purified protein (Figure 8A), located in the outer edge of the drop. Component 3 is representative of the noise. In total, almost all the information was extracted (Q residuals of approximate %). In general, the coffee ring is observed where the buffer salts and excipients evaporated around the center of the drop, and the protein molecules moved outward to the edge and concentrated on the outer ring of the drop. This contact line pinning acts as a barrier, thus, limiting any further spread of the solvent at the edge. The capillary flow in the drop is from the center of the drop towards the line of contact because the evaporation flow at the periphery is greater than at the center of the drop. Thus, to compensate for the greater loss of solvent at the level of the line of contact, the solvent flow starting from the center is directed towards the line of contact with the non-volatile solutes. Thus, the local evaporation rate is considered to be an important factor in order to ensure the efficiency of the separation between the excipients and the protein [24,25]. 

The phenomenon of the formation of coffee ring patterns in drying drops can also depend on drying conditions, such as temperature, air velocity, and relative humidity in the evaporating medium. Relative humidity directly influences the contact angle of the drop and, thus, the initial evaporation rate of the drop. Indeed, their experiments showed that the contact angle decreases with relative humidity, which influences the pattern of coffee ring formation at the end of the evaporation process [26].

The differential solubilities in the various components in the solution also play an important role in segregation. Proteins precipitate at the beginning of the evaporation process, whereas highly soluble compounds, such as buffer solutions or compounds present at very low initial concentration, may remain dissolved in the drop, which evaporates for much longer, and, therefore, tends, eventually, to settle at the center of the drop [10,24].

Further, a question is raised in this part: is it possible to identify the dominant component (Table S1) among the excipients for each mAbs? Therefore, superposition of each excipient was detected by the MCR-ALS in the center component for each mAbs, with each of the excipients present in the formulation of the mAbs. It is interesting to note that the spectrum of excipient found in the center of the drop corresponds to the dominant excipient (at the highest concentration) in the formulation of the mAbs (Table S1). These same excipients were retained in the filter of the Microbiospin^®^ column, during the purification of the mAbs in Table 2.

To evaluate the MCR-ALS model quality, the percentage of lack of fit (LOF) was calculated. Therefore, the values of lack of fit, after applying the SVD, were close to zero (Table S2), indicating a good quality for the matrix reconstruction. It can be deduced that the analysis of the mAbs by DCDR imaging associated with the MCR-ALS is an efficient technique to eliminate the interference of the excipients. The separation of proteins and excipients by physical segregation plays the same role as purification based on Microbiospin^®^ column by concentrating the protein at the periphery and the excipients at the center.

The DCDR method can be used to track the progress of wash/ultrafiltration cycles used to remove impurities and additives, such as glycerol and buffer compounds. Indeed, despite an optimization in the number of washing cycles, it happens that certain excipients are not completely eliminated [6]. Additionally, as described in previous studies, this method can be used to detect protein impurities [27].

### 3.3. Limit of Detection of DCDR Analysis

The study on detection limit was performed, in order to evaluate the efficiency of the separation process of the DCDR method, because it turns out that this method is more efficient at low concentration. Figure S2 shows the images of the drops corresponding, respectively, to each dilution of trastuzumab and Figure S3 shows the evolution of the intensity of the bands for the excipients and the protein in the center and at the edge of the drop.

The presence of protein signals in the center of the drop at high concentration shows that the segregation of proteins is incomplete due to the high protein content in a small volume of solvent that evaporates quickly, leaving insufficient time for the protein to move completely to the periphery. In addition, according to Figure S3, as the concentration of mAbs decreases, agglomerates of excipients are more concentrated and form in the center and the protein concentrates at the edge of the drop. This is due to the fact that the more the formulation of mAb is diluted, the more the excipients have time to regroup and form agglomerates and the more the proteins have time to settle entirely at the edge. Thus, the separation between the excipients and the protein within the DCDR method is more efficient at low concentration. The detection limit for the 16 mAbs is around 0.5 mg mL^−1^ according to the S/N ratio.

The question of the repeatability of DCDR spectra can be raised. Studies on protein analysis have already shown that the repeatability of DCDR spectra can depend on the thickness of the protein cycle but also on the purity of the solvent. For example, the independent deposit of different volumes of the same solution (100 and 1 μM) generates a difference in thickness of the protein rings for the two deposits. Thus, the signal-to-noise ratios for the deposition spectrum in the low volume are significantly higher than these at larger volume. However, they clearly demonstrated that in the case of independent deposition of the same volume in the same solution, there is no significant spectral variation [27].

### 3.4. SVR Models to Determine the Composition of Monosaccharides and Glycans

In this study, the reference data were obtained via UPLC-FLR-MS. The mass spectrometry data were used to identify N-glycans and the fluorescence data were used for glycan quantification. Table S3 groups the composition of the main N-glycans for each mAbs and Table S4 presents the overall mass percentage of the five monosaccharides present in each mAb [1]. 

The SVR regression models were built to predict the amount of each monosaccharide (mannose, N-acetylglucosamine, galactose, fucose, sialic acid) and glycans, using the spectra of pure component of the glycoprotein (Figure S4) extracted from the MCR-ALS. Optimized SVR parameters for monosaccharide and glycan models at low concentration are presented in Tables S5 and S6.

**Figure 9:**
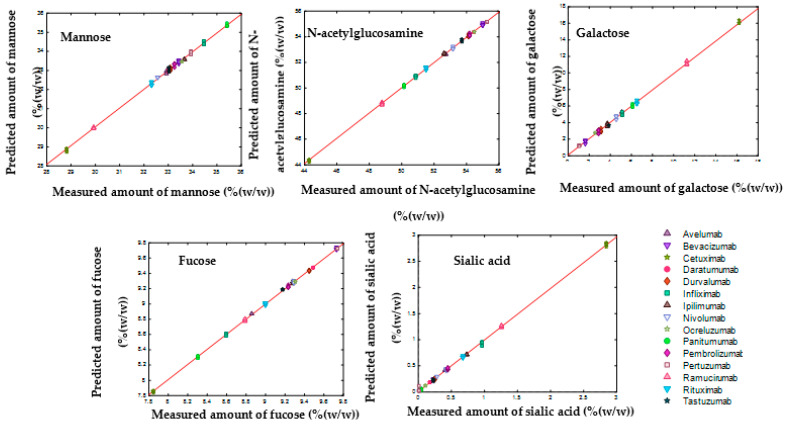
Measured versus predicted amounts of monosaccharides obtained by SVR regression between 800 and 1150 cm^−1^ for the analysis of the amount of each monosaccharide at low concentration (3 mg mL^−1^) of mAbs.

**Figure 10:**
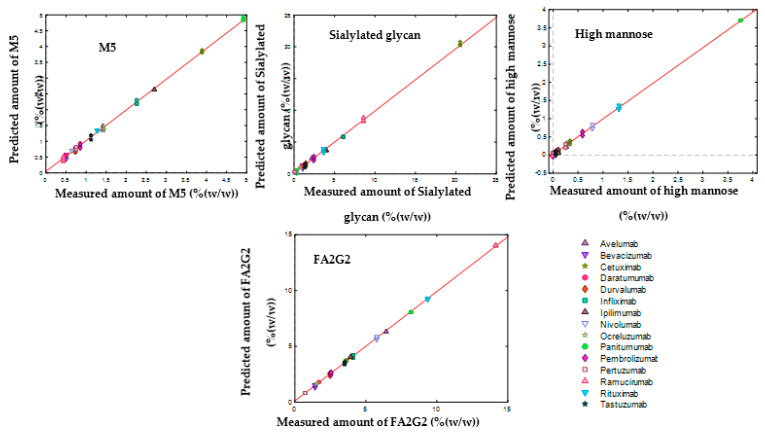
Measured versus predicted amounts of monosaccharides obtained by SVR regression between 800 and 1150 cm^−1^ for the analysis of the amount of each glycan at low concentration (3 mg mL^−1^) of mAbs.

Table 3 and Table 4 demonstrate the performances of SVR models in predicting the amount of each monosaccharide and glycan and Figure 9 and Figure 10 show the results of data modelling by the SVR model, respectively. These results show low error values in terms of calibration (RMSEC), cross-validation performance (RMSECV), and high $R_{cv}^{2}$ values, indicating that the models captured most of the correlation between the spectral data and the quantitative values. 

### 3.5. Interpretation SVR Models

**Figure 11:**
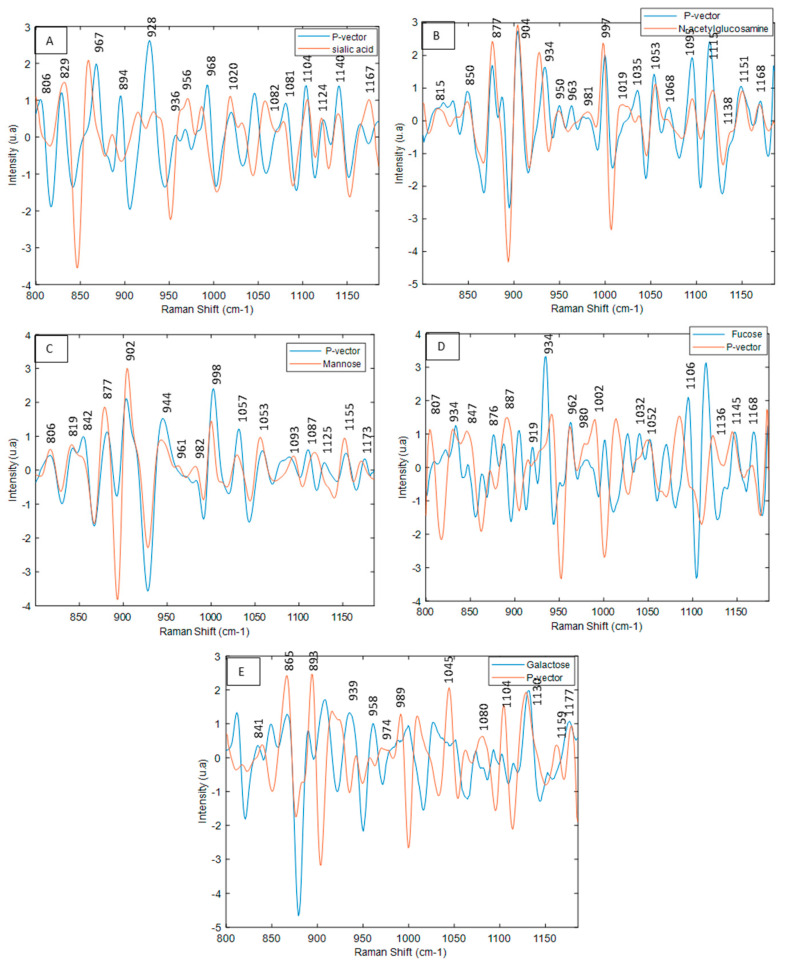
Superimposition of the P-vector with each respective monosaccharide. (**A**) Sialic acid SVR model. (**B**) N-acetylglucosamine SVR model. (**C**) Mannose SVR model. (**D**) Fucose SVR model. (**E**) Galactose SVR model.

Unlike the PLSR of which loadings can be interpreted, SVR provides less diagnostics on the model in terms of specificity. In an SVR model, the information related to the original input variables is lost. Therefore, to study the specificity of the SVR models, P-vectors of the different models were investigated. The P-vectors show the contribution of each input variable (original data) to the final regression model. Figure 11 shows the results of the monosaccharide models. In the mannose model (Figure 11C), all mannose characteristic bands (1180, 1156, 1124, 1081, 1048, 992, 967, 945, 900, 870, 848, 821, 808 cm^−1^) are present in the P-vector (build in SVR). The same results were observed for the sialic acid, N-acetylglucosamine models (Figure 11A,B). With regards to the P-vector of the fucose and galactose model (Figure 11D,E), the main bands characteristic of the monosaccharides are also found; however, there are slight differences in some bands. This is partially explained by the existence of different environments surrounding the chemical bonds of monosaccharides in glycoproteins (different protein sequences implying different conformations), implying minor differences in the vibrations [1]. 

**Figure 12:**
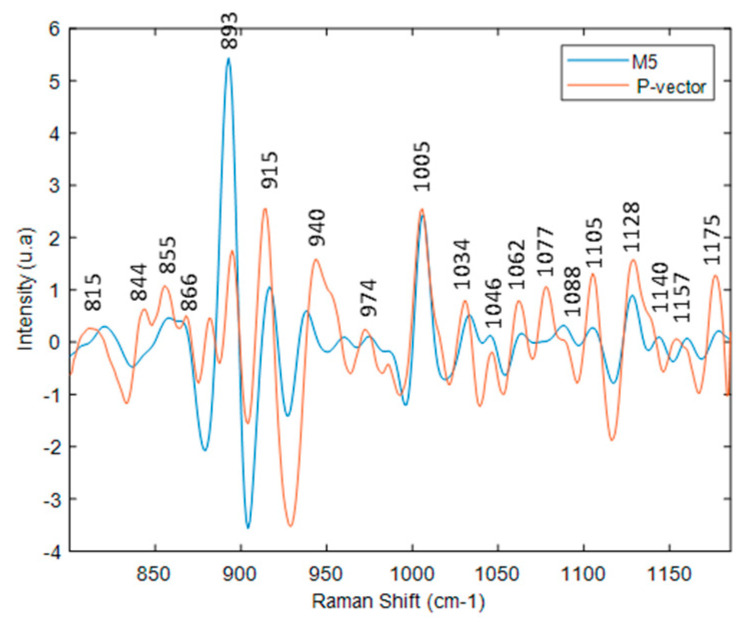
Superposition of the P-vector SVR model with M5 glycan.

For the M5 glycan model (Figure 12), all M5 glycan characteristic bands (1170, 1155, 1142, 1128, 1105, 1089, 1077, 1062, 1050, 1038, 1009, 978, 935, 915, 894, 863, 847, 820 cm^−1^) are present in the P-vector. 

## 4. Conclusions

Glycosylation is one of the most critical attributes of biopharmaceuticals to be monitored from development to production. In previous work, we suggested using FT-IR spectroscopy in ATR mode to characterize and quantify glycosylation and, thus, determine the composition of monosaccharides in glycoproteins. This approach has many advantages: reduced sample preparation (no cleavage, labelling, or separation step) and concise measurement time. However, this method has some limitations; for example, filtration columns were used to remove the excipients, which can interfere with the FT-IR measurements in the specific region of carbohydrate signals. Additionally, it requires protein deposits with a uniform chemical composition. This may be difficult or impossible to achieve due to the coffee ring effect, which concentrates the protein in a ring, as well as the phenomenon of segregation, which tends to cause different components in the solution to precipitate at different locations on the substrate. In this study, it is all to our advantage, due to the DCDR benefiting enormously from the effects of the coffee ring [27]. This is why, in order to simplify the sample preparation step and to increase the analysis throughput, we propose here using a fast and direct protocol based on a drop-coating deposition Raman imaging (DCDR) method combined with multivariate curve resolution alternating least square (MCR-ALS) analysis and SVR regression to identify and quantify glycosylation in monoclonal antibodies (mAbs).

First, DCDR imaging combined with MCR-ALS analysis were applied to 16 mAbs to demonstrate the ability of the method to extract pure Raman spectra of glycoproteins present in their original pharmaceutical formulation. The process of drying the drop allowed the separation of compounds in the solution by forming a “coffee ring” [2]. It was shown that, while the buffer salts and excipients evaporated around the center of the drop, the proteins moved outward to the edge and concentrated on the outer part of the drop. By evaluating the MCR-ALS model quality by LOF, the results show that the values of lack of fit, before applying the SVD, were go until 14%, while after applying the SVD, they were close to 0%. It can be deduced that the analysis of the mAbs by DCDR imaging associated with the MCR-ALS and SVD is an efficient technique to eliminate the interference of the excipients.

The second part of the study demonstrates the capacity of DCDR spectroscopy to quantify the relative amount of each monosaccharide and glycan. In this context, the SVR regression models were established on the spectral region of glycans, between 800 and 1150 cm^−1^. The SVR models of each monosaccharide and glycan exhibited good analytical calibration performances. Indeed, low values of RMSEC and RMSECV indicate high accuracy and high value of $R_{CV}^{2}$ close to 1, indicating that the model correctly handles the spectral variability and is, therefore, able to accurately estimate the concentration.

The specificity of SVR models was also evaluated. It can be concluded that this interpretation approach facilitates the understanding and in-depth analysis of SVR models. 

Finally, the results presented so far prove the concept of this approach for quantification of monosaccharides and glycans in mAbs and, thus, pave the way to three potential applications: comparing the glycosylation of a biosimilar and the original molecule, monitoring batch-to-batch homogeneity, and in-process control of glycoprotein bioproduction.

## Figures and Tables

**Table 1:** The different preparations of dilutions of the 16 mAbs.

Initial Concentration (mg mL^−1^)	Dilution Range (mg mL^−1^)
5	4, 3, 2, 1, 0.5, 0.1, 0.01
10	7, 5, 3, 1, 0.5, 0.1, 0.01
20	10, 5, 3, 1, 0.5, 0.1, 0.01
21	10, 5, 3, 1, 0.5, 0.1, 0.01
25	12.5, 5, 3, 1, 0.5, 0.1, 0.01
30	15, 5, 3, 1, 0.5, 0.1, 0.01
50	25, 5, 3, 1, 0.5, 0.1, 0.01

**Table 2:** Excipient list detected in the analyzed commercial mAbs.

mAbs	Excipient Detected
Pembrolizumab Infliximab Pertuzumab	Sucrose
Cetuximab Ramucirumab	Glycine
Ocreluzumab Durvalumab Trastuzumab Bevacizumab	Trehalose
Avelumab Daratumumab Nivolumab Ipilimumab	Mannitol
Panitumumab	Sodium acetate
Rituximab	Sodium citrate

**Table 3:** Performances of SVR models in predicting monosaccharides at low concentration.

	Mannose	N-acetylglucosamine	Galactose	Fucose	Sialic Acid
Number of support vectors	60	70	72	71	66
R^2^_Cal_	1.00	1.00	1.00	1.00	1.00
R^2^_CV_	1.00	0.95	0.96	0.84	0.86
RMSEC (%(*w*/*w*))	0.08	0.07	0.18	0.01	0.05
RMSECV (%(*w*/*w*))	0.72	1.05	0.85	0.23	0.34

**Table 4:** Performances of SVR models in predicting glycan at low concentration.

	M5	Sialylated Glycan	High Mannose	FA2G2
Number of support vectors	62	62	64	68
R^2^_Cal_	1.00	1.00	1.00	1.00
R^2^_CV_	0.52	0.95	0.83	0.88
RMSEC (%(*w*/*w*))	0.06	0.25	0.04	0.11
RMSECV (%(*w*/*w*))	0.92	1.80	0.58	1.30

## Data Availability

Not applicable.

## Supplementary Materials

The following supporting information can be downloaded at: https://www.mdpi.com/article/10.3390/molecules27144405/s1, Figure S1. (A) Trastuzumab filtrate (trehalose) drop image acquired with the 10× objective (B) filter spectrum (C) superposition of the spectrum of the filter with the excipient trehalose; Figure S2. Image of the drops acquired at different concentrations (21, 10, 5, 3,1, 0.5, 0., 0.01 mg/mL) for trastuzumab analysis; Figure S3. Evolution of the intensity displayed in color code, according to the intensity of the information, from the least intense (blue color) to the most intense (yellow color). The bands of the excipient (849, 1358 cm^−1^) and of the protein (1004, 1673 cm^−1^), at the edge (A) and in the center (B) of the drop were evaluated; Figure S4: Raman spectra (principal component extracted in MCR-ALS) preprocessed by the baseline correction, in the spectral range between 800 and 1150 cm^−1^ for the analysis of the composition of monosaccharides and glycans; Table S1: Excipients list present in the analyzed commercial solution of mAbs; Table S2: Percentage of lack of fit (LOF); Table S3: Mass percentage of the majority of N-glycans for each of the glycoproteins. This percentage was calculated based on relative peak areas-%RPA using UPLC-FLR-MS analysis of N-glycans after they were released. labelled and purified by GlycoWorks RapiFluor-MS N-glycan kit from Waters; Table S4: Overall mass percentage of the 5 monosaccharides present in each glycoprotein. These results were obtained by calculating the relative peak areas %RPA; Table S5: Optimal parameters in SVR model for predicting the composition of monosaccharides in low concentration; Table S6: Optimal parameters in SVR model for predicting the composition of glycans in low concentration.

## Sample Availability

Samples of the compounds are not available from the authors.
