# Supplementary material for: A New Alternative Tool to Analyse Glycosylation in Monoclonal Antibodies Based on Drop-Coating Deposition Raman imaging: A Proof of Concept
Source: Molecules. 2022 Jul 9;27(14):4405. doi: 10.3390/molecules27144405 (PMC9317070; doi:10.3390/molecules27144405)
Supplement: Supplementary file 1 [file molecules-27-04405-s001.zip › molecules-1786283-supplementary.pdf]

Electronic Supplementary information (ESI)

A new alternative tool to analyse glycosylation in monoclonal antibodies based on drop-coating deposition Raman imaging : A proof of concept

Sabrina Hamla[1*], Pierre-Yves Sacré[1], Allison Derenne[2], Ben Cowper[3], Erik Goormaghtigh[2], Philippe Hubert[1], Eric Ziemons[1].

1 University of Liege (ULiege), CIRM, Vibra-Sante Hub, Department of Pharmacy, Laboratory of Pharmaceutical Analytical Chemistry, Liege, Belgium

2 Center for Structural Biology and Bioinformatics, Laboratory for the Structure and Function of Biological Membranes, ULB, Campus Plaine CP206/02, 1050 Brussels, Belgium

3 National Institute for Biological Standards and Control, Blanche Lane, South Mimms, Potters Bar, Hertfordshire, UK

Spectral characterisation of commercial mAbs solutions after removal of excipients by Microbiospin

Analysis of the excipients

The image (A) of the drop of trastuzumab, it shows the formation of agglomerates in the center, which corresponds to the excipient trehalose.

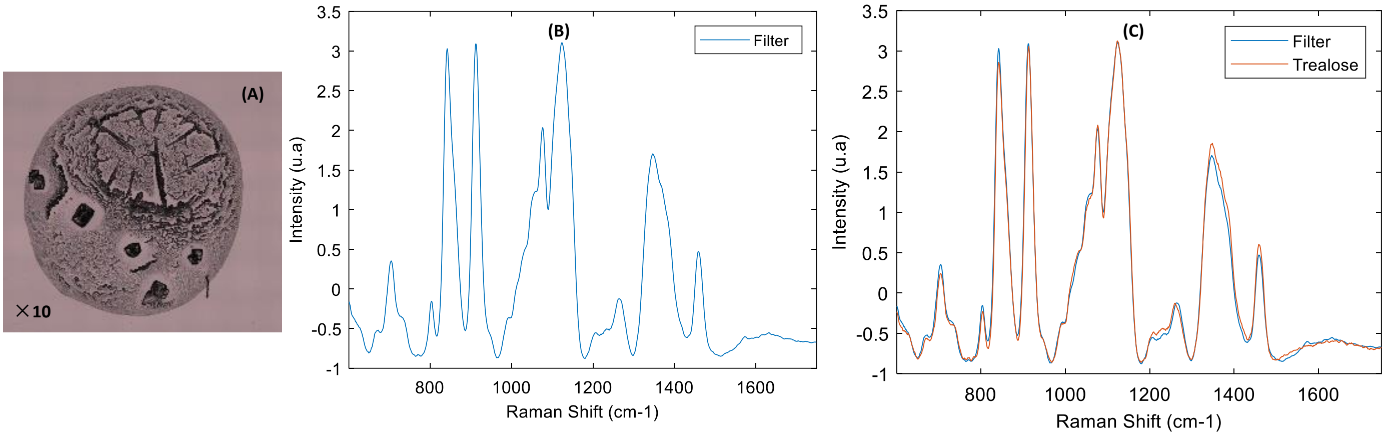

Figure S1. (A) Trastuzumab filtrate (trehalose) drop image acquired with the 10x objective (B) Filter spectrum (C) Superposition of the spectrum of the filter with the excipient trehalose.

Table S1: Excipients list present in the analyzed commercial solution of mAbs

Brand	mAbs	Excipients (mg mL^{-1})
**Darzalex®**	Daratumumab	Mannitol: 25<br>Polysorbate 20: 0.4<br>Acetic acid: 0.185<br>Sodium acetate: 2.9<br>Sodium chloride: 3.5
**Perjeta®**	Pertuzumab	Sucrose:120<br>Polysorbate 20: 0.4<br>histidine acetate: 0.020
**Ocrevus®**	Ocreluzumab	Trehalose: 40<br>Polysorbate 20: 0.2<br>Acetic acid: 0.25<br>Sodium acetate: 2.14
**Vectibix®**	Panitumumab	Sodium chloride: 0.10<br>Sodium acetate: 6.6
**Cyramza®**	Ramucirumab	Polysorbate 80: 0.1<br>Glycine: 9.98<br>L-histidine: 0.65<br>L-histidine hydrochloride monohydrate: 1.22<br>Sodium chloride: 4.4

**Opvido®**	Nivolumab	Sodium chloride: 2.92<br>Mannitol: 30<br>Pentetic acid: 0.008<br>Polysorbate 20: 0.2<br>Sodium citrate: 5.88
**Imfinzi®**	Durvalumab	Trehalose: 140<br>Polysorbate 80: 0.2<br>L-histidine: 2<br>L-histidine hydrochloride monohydrate: 2.7
**Keytruda®**	Pembrolizumab	Sucrose: 70<br>Polysorbate 80: 0.2<br>L-histidine: 1.55
**Remicade®**	Infliximab	Sucrose: 50<br>Polysorbate 80: 0.05<br>Sodium phosphate: 0.006
**Yevroy®**	Ipilimumab	Mannitol: 10<br>Sodium chloride: 5.85<br>Pentetic acid: 0.04<br>Polysorbate 80: 0.1<br>Tromethamine HCL: 3.15
**Mabthera®**	Rituximab	Sodium chloride: 9<br>Polysorbate 80: 0.7<br>Sodium citrate: 7.35
**Herceptin®**	Trastuzumab	Trehalose dihydrate: 20<br>Polysorbate 20: 0.08<br>L-histidine hydrochloride monohydrate: 0.49<br>L-histidine: 0.32
**Bavencio®**	Avelumab	Mannitol: 51<br>Polysorbate 20: 0.5<br>Acetic acid: 0.6
**Avastin®**	Bevacizumab	Trehalose dihydrate: 60<br>Sodium phosphate: 0.05<br>Polysorbate 20: 0.4<br>Water for injections.
**Erbitux®**	Cetuximab	Sodium chloride: 5.8<br>Glycine: 7.5<br>Polysorbate 80: 0.1<br>Citric acid: 2.1<br>monohydrate Sodium hydroxide:<br>Water for injections.

Spectral characterization of commercial mAbs solutions by DCDR imaging and MCR-ALS analysis

Table S2: Percentage of lack of fit (LOF)

mAbs	LOF (%)	LOF (%) after application of SVD
**Avelumab**	2.30	0.04
**Bevacizumab**	3.78	0.01
**Cetximab**	7.77	1.01
**Daratumumab**	4.12	0.33
**Durvalumab**	14.35	0.77
**Infliximab**	3.09	0.11
**Ipilimumab**	4.22	0.28
**Nivolumab**	13.47	1.25
**Ocreluzumab**	2.10	0.87
**Panitumumab**	6.57	0.27
**Pembrolizumab**	2.76	0.00
**Pertuzumab**	4.83	0.68
**Ramucirumab**	7.6	0.35
**Rituximab**	8.12	0.21
**Trastuzumab**	7.42	0.09
**Ustekinumab**	5.7	0.85

Limit of detection of DCDR analysis

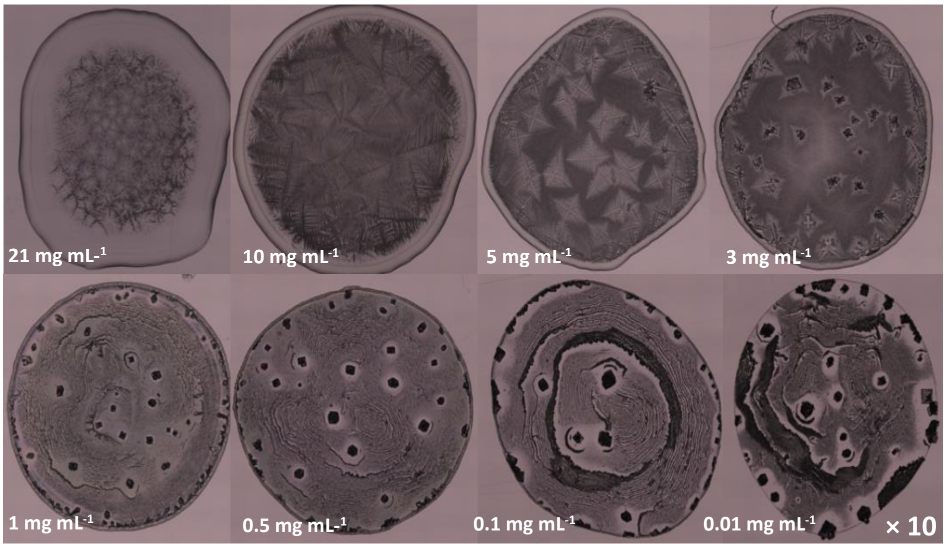

Figure S2. Image of the drops acquired at different concentrations (21, 10, 5, 3,1, 0.5, 0., 0.01 mg mL^{-1}) for trastuzumab analysis.

By following the two bands of trehalose (main excipient) at 849 and 1358 cm^{-1}, the intensity of these two bands decreases for diluted solutions at the edge of the drop and increases in the center. It is the opposite for the two protein bands at 1004 and 1673 cm^{-1}.

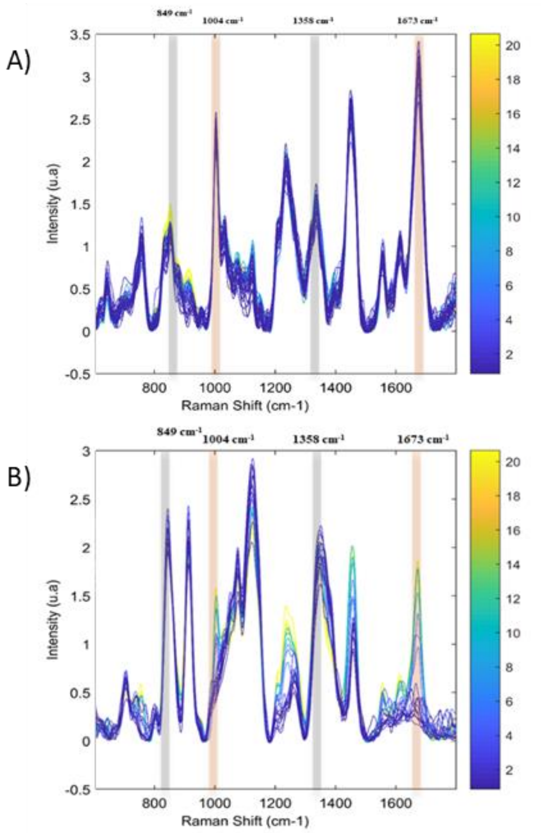

Figure S3. Evolution of the intensity displayed in color code from the least intense (blue color) to the most intense (yellow color). The bands of the excipient (849 and 1358 cm^{-1}) and of the protein (1004 and 1673 cm^{-1}) at the edge (A) and in the center (B) of the drop were evaluated.

Comparison of the performances of PLSR and SVR to determine the composition of monosaccharides and glycans

Table S3: Mass percentage of the majority of N-glycans for each of the glycoproteins. This percentage was calculated based on relative peak areas-%RPA using UPLC-FLR-MS analysis of N-glycans after they were released. labelled and purified by GlycoWorks RapiFluor-MS N-glycan kit from Waters.

	FA2 (%(w/w))	FA2G1 (%(w/w))	FA2G2 (%(w/w))	M5 (%(w/w))	High mannose (M6. M7. M8) (%(w/w))	Sialylated glycans (%(w/w))	α-Gal- Gal-containing glycans (%(w/w))
**Avelumab**	48.92	34.00	6.43	1.42	0.27	2.19	0.00
**Bevacizumab**	79.92	11.49	1.41	0.51	0.00	1.07	0.00
**Cetuximab**	18.64	18.51	3.63	3.89	0.34	20.52	46.49
**Daratumumab**	70.03	23.79	1.68	0.51	0.09	1.00	0.00
**Durvalumab**	67.71	23.98	2.47	0.73	0.05	1.44	0.00
**Infliximab**	44.30	27.67	4.09	2.26	0.00	6.07	2.37
**Ipilimumab**	59.20	23.02	3.94	2.70	0.10	3.97	0.00
**Nivolumab**	56.66	30.24	5.76	0.64	0.79	2.35	0.00
**Ocrelizumab**	69.59	20.59	1.54	1.45	0.00	0.71	0.00
**Panitumumab**	35.58	39.35	8.18	4.91	3.75	0.30	0.00
**Pembrolizumab**	67.84	19.70	2.54	0.85	0.59	2.44	0.00
**Pertuzumab**	83.21	8.69	0.72	0.76	0.25	0.06	0.00
**Ramucirumab**	20.03	43.25	14.15	0.43	0.00	8.57	14.25
**Rituximab**	40.29	41.13	9.36	1.28	1.32	3.65	0.00
**Trastuzumab**	57.84	28.39	3.50	1.12	0.05	1.32	0.00
**Ustekinumab**	26.78	35.28	6.96	0.60	0.00	25.19	5.48

Table S4: Overall mass percentage of the 5 monosaccharides present in each glycoprotein. These results were obtained by calculating the relative peak areas %RPA.

	Mannose (%(w/w))	N-acetylglucosamine (%(w/w))	Galactose (%(w/w))	Fucose (%(w/w))	Sialic Acid (%(w/w))
**Avelumab**	32.92	52.72	5.09	8.86	0.40
**Bevacizumab**	33.43	54.99	1.62	9.73	0.23
**Cetuximab**	28.82	44.29	16.20	7.85	2.84
**Daratumumab**	33.01	54.44	2.88	9.49	0.18
**Durvalumab**	33.08	54.12	3.11	9.45	0.25
**Infliximab**	34.46	50.86	5.12	8.59	0.96
**Ipilimumab**	33.66	52.61	3.72	9.28	0.73
**Nivolumab**	32.56	53.16	4.57	9.29	0.42
**Ocrelizumab**	33.55	54.47	2.56	9.31	0.11
**Panitumumab**	35.41	50.14	6.10	8.30	0.05
**Pembrolizumab**	33.25	54.19	2.88	9.23	0.45
**Pertuzumab**	33.93	55.27	1.06	9.74	0.01
**Ramucirumab**	29.93	48.78	11.25	8.79	1.26
**Rituximab**	32.31	51.50	6.52	9.00	0.67
**Trastuzumab**	33.02	53.71	3.87	9.18	0.23
**Ustekinumab**	29.91	49.01	8.78	8.37	3.94

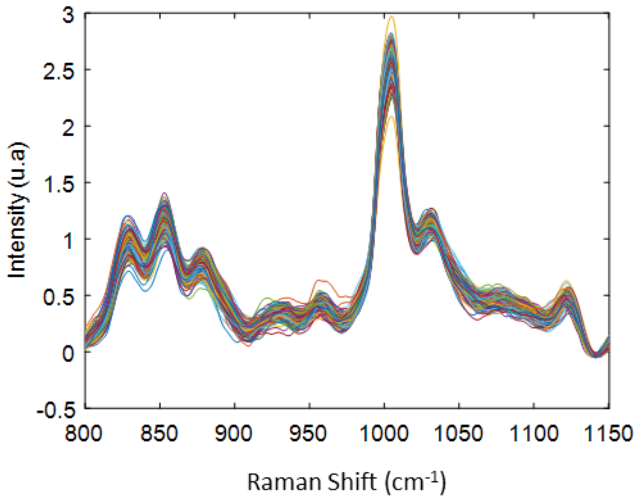

Figure S4: Raman spectra of 16 mAbs (principal component extracted in MCR-ALS) preprocessed by the baseline correction, in the spectral range between 800 and 1150 cm^{-1} for the analysis of the composition of monosaccharides and glycans.

Table S5: Optimal parameters in SVR model for predicting the composition of monosaccharides in low concentration (3mg mL^{-1}).

		Optimal SVR parameters		
		γ	ε	C
**Model monosaccharides**	Galactose	0.01	0.05	100
	Mannose	0.01	0.05	10
	Fucose	0.01	0.025111	100
	Sialic acid	0.01	0.02	6
	N-acetylglucosamine	0.01	0.03	100

Table S6: Optimal parameters in SVR model for predicting the composition of glycans in low concentration (3mg mL^{-1}).

		Optimal SVR parameters		
		γ	ε	C
**Model glycans**	M5	0.01	0.05	50
	Sialylated glycan	0.01	0.05	50
	High mannose	0.01	0.05	50
	FA2G2	0.0316<br>23	0.03	6
